# Paraquat poisoning induced pulmonary epithelial mesenchymal transition through Notch1 pathway

**DOI:** 10.1038/s41598-017-01069-9

**Published:** 2017-04-19

**Authors:** Tiegang Li, Xiangming Yang, Shiyu Xin, Yan Cao, Nana Wang

**Affiliations:** 1 Emergency Department, Shengjing Hospital of China Medical University, Shenyang, 110004 China; 2 Emergency Department, The Fourth Hospital of China Medical University, Shenyang, 110004 China; 3 Endocrinology Department, Shengjing Hospital of China Medical University, Shenyang, 110004 China

## Abstract

Progressive pulmonary fibrosis is the most characteristic feature of subacute PQ poisoning. Epithelial-to-mesenchymal transition (EMT) is reported to be involved in the pulmonary fibrosis after PQ exposure. Recent evidence suggested Notch signaling is required for EMT. In this study, we investigated whether Notch1 and TGF-β1/Smad3 signaling was involved in EMT caused by PQ. It is demonstrated that A549 cells underwent EMT after treated with PQ at dose of 300 μmol/L for 6 days, charactered by increasing expression of mesenchymal marker α-SMA and decreasing expression of epithelial marker E-cadherin. We found that there was an apparent increased expression of Notch1 and jagged-1 in PQ induced EMT process. EMT could be enhanced by Jagged-1 ligand of Notch1, and be blocked by DAPT, a γ-secretase inhibitor. Our data also showed that the expression of TGF-β1/Smad3 increased after Notch1 is elevated in EMT caused by PQ. Jagged-1 significantly induced SMA expression, and this induction was completely inhibited by SB431542 in A549 cells. In conclusion, we demonstrated that Notch1 pathway was important in EMT induced by PQ, and TGF-β1/Smad3 signaling partly plays a role as the downstream of Notch1.

## Introduction

Paraquat (PQ, 1,1′-dimethyl-4,4′-bipyridinium), widely used as a herbicide in many developing countries around the world, can cause severe toxicity in human and animals. It is reported that PQ poisoning accounts for up to a third of all suicides worldwide^1^. After PQ poisoning accidentally or intentionally, patients develop acute multi-organ failure, followed by progressive pulmonary fibrosis and eventually death from respiratory failure. Progressive pulmonary fibrosis is the most characteristic feature of subacute PQ poisoning, which occurs over a period of days to weeks after PQ ingestion^2^. Although PQ-induced pulmonary fibrosis is associated with high mortality, the molecular mechanism of its toxicity is not yet established, and there are no effective antidotes available. It is widely understood that pulmonary fibrosis is a result of the proliferation of fibroblasts from profibroblasts and the subsequent accumulation of extracellular matrix proteins^3^. However, recent research has demonstrated that mesenchymal stem cells derived from the bone marrow^4, 5^, and fully differentiated alveolar epithelial cells were capable of undergoing phenotypic switching to fibroblasts through a process termed the “epithelial-to-mesenchymal transition” (EMT)^6^.

EMT is a process in which the polarized immotile epithelial cells are converted to motile mesenchymal cells through molecular reprogramming and phenotypic changes. Under physiological and pathological conditions, EMT is involved in the remodeling of tissues during embryonic development, tumor progression and development of fibrosis. During this transition, epithelial cells acquire the capacity to increase motility through down-regulating epithelial markers, such as E-cadherin, and up-regulating mesenchymal proteins, such as α-SMA^7–9^. Recent studies suggest the potential contributions of EMT in lung fibrosis^10–12^. Therefore, it is indeed possible that EMT may play an important role in pulmonary fibrosis caused by PQ poisoning. Recently, a study by Yamada *et al*. demonstrated the occurrence of EMT in lung fibrosis induced by PQ^13^.

Previous reports indicated that Notch-mediated cell-to-cell signaling regulates development by controlling cell fate determination, cell proliferation, differentiation, and apoptosis during embryonic and postnatal stages^14, 15^. Notch proteins are transmembrane receptors (Notch 1–4). Notch signaling is initiated by the interaction between the ligand (delta/jagged family) on one cell and the receptor on a neighboring cell. After ligand binding, γ-secretase-dependent proteolytic cleavage of the Notch receptor is triggered to release the Notch intracellular domain (NICD) to the nucleus which then activates target genes^16, 17^.

Recent evidence suggested that Notch signaling is involved in the EMT process. It has been reported that EMT is upregulated in fibrotic diseases of organs such as kidney, liver, and lungs *in vivo* ^18–20^. Overexpression of NICD in podocytes can promote glomerular fibrosis in transgenic mice. Utilizing GSI, a γ-secretase inhibitor, relieved kidney fibrosis in mice^21^. In pulmonary fibrosis, activation of Notch pathway in alveolar cells could elevate expression of ACTA2 and COL1A1, and proliferation to myofibroblast^22^. Furthermore, blocking of Jagged1/Notch pathway by RNA interference, could effectively inhibit the switching from keratinocyte to mesenchymal cell^23^. Present investigations demonstrated that EMT induced by Notch signaling related to TGF-β, and TGF-β acts as the downstream of Notch signaling^24^.

In the present study, we investigated whether Notch1 pathway is involved in EMT caused by PQ poisoning. Our data, for the first time, showed that Notch1 pathway was upregulated in EMT process after PQ poisoning in A549 cells in an *in vitro* cell culture model. Moreover, transforming growth factor-β (TGF-β1) was enhanced, and EMT was accelerated, after Jagged1 ligand overexpression in PQ poisoning. We also demonstrated that Smad-dependent signaling pathway was activated in response to PQ poisoning. These findings suggest that PQ poisoning active the Notch1 pathway by up-regulating the expression of TGF-β/Smad3, which resulted in the process of EMT.

## Results

### PQ induced EMT-like phenotypic changes in A549 cells

**Figure 1:**
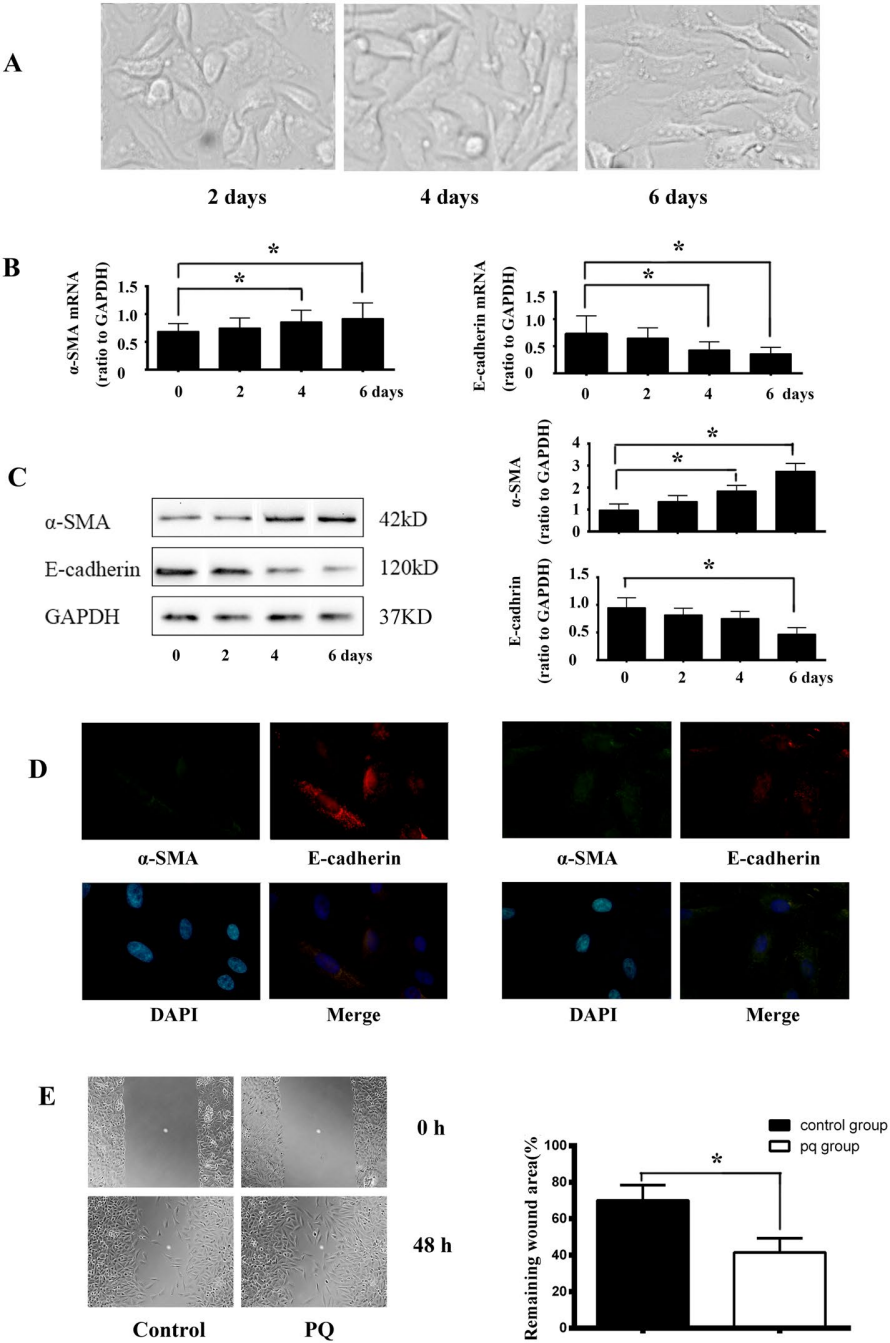
PQ induced EMT-like phenotypic changes in A549 cells. (**A**) Morphological changes of A549 cells induced by PQ exposure (300 μmol/L) at different time points. After exposure to PQ, the cells gradually displayed morphological appearances of mesenchymal cells and after 6 days of PQ exposure, the majority of cells became spindle-shaped. (**B**) Quantitative RT-PCR analysis result of α-SMA and E-cadherin mRNA in A549 cells after exposure to PQ at indicated time points. PQ treatment induced increasing of mRNA of α-SMA and decreasing of E-cadherin after 4 days of exposure. The qRT-PCR results shown represent an average of three independent experiments. (**C**) Alterations of α-SMA and E-cadherin proteins expression in PQ treated A549 cells in serials of time by Western blot analysis. A dramatic decrease of E-cadherin protein was detected on the 6th days and an increase of α-SMA protein level on the 4th day after PQ exposure. Representative blots from three independent experiments are shown. Density of α-SMA and E-cadherin were quantified and expressed as the ratio to GAPDH. (**D**) Increased cell membrane distribution ofα-SMAprotein and decreased E-cadherin proteininA549cells in respond to PQ stimulation as assessed by immunofluorescent staining. α-SMA was stained green, E-cadherin was stained red and DAPI stained nuclei blue. (**E**) A significantly increased migration capacity after PQ exposure by about 0.41 fold was calculated than that of normal A549 cellsby monolayer wound-healing assay. *p < 0.05.

To investigate whether PQ could induce EMT in A549 cells, we exposed cells to different concentrations of PQ with different time in a preliminary experiment (not shown). We identified 300 μmol/L as the concentration at which EMT could occur with minimum toxicity to cells. So, in the subsequent experiments we used PQ at a concentration of 300 μmol/L. In the absence of PQ, A549 cells maintained epithelial morphology during the culture period. After exposure to PQ, the cells gradually displayed morphological appearances of mesenchymal cells. And after six days of PQ exposure, the majority of cells became spindle-shaped (Fig. 1A). These morphological features suggested that PQ may induce EMT-like phenotypic changes in A549 cells.

During EMT process, epithelial cells acquire the capacity to increase motility through down-regulation of epithelial markers, such as E-cadherin, and up-regulation of mesenchymal proteins, such as α-SMA. The increase in α-SMA and decrease in E-cadherin expression is a marker of the occurrence of EMT. We found that after four days of PQ treatment, α-SMA mRNA levels were statistically increased in A549 cells as compared to day zero by qRT-PCR analysis. Meanwhile, E-cadherin mRNA levels were dramatically decreased after four days of PQ exposure (Fig. 1B).

Western blot analysis revealed a similar decrease in E-cadherin and increase in α-SMA protein level in PQ-treated A549 cells, which correlated with mRNA changes. Specifically, PQ treatment for four days induced only a modest decrease in E-cadherin protein, while a dramatic decrease of E-cadherin protein was detected six days after PQ exposure (Fig. 1C).

Decreased expression of E-cadherin and increased expression of α-SMA in PQ treated A549 cells were further confirmed by immunofluorescent staining. As shown in Fig. 1D, control A549 cells cultured in the absence of PQ predominantly expressed E-cadherin on the cell membrane, whereas cells in the presence of PQ gradually lost E-cadherin expression and increased expression of α-SMA.

EMT induction in cells results in the acquisition of migration property. To evaluate the migration potential of A549 cells, a wound-healing assay was performed. The results showed a significantly increased migration capacity after PQ exposure by about 0.41 fold than that of normal A549 cells (Fig. 1E).

All the data together revealed that A549 underwent EMT after PQ exposure at a dose of 300 μmol/L for six days.

### Notch1 signaling is required for PQ-induced EMT-like changes in A549 cells

**Figure 2:**
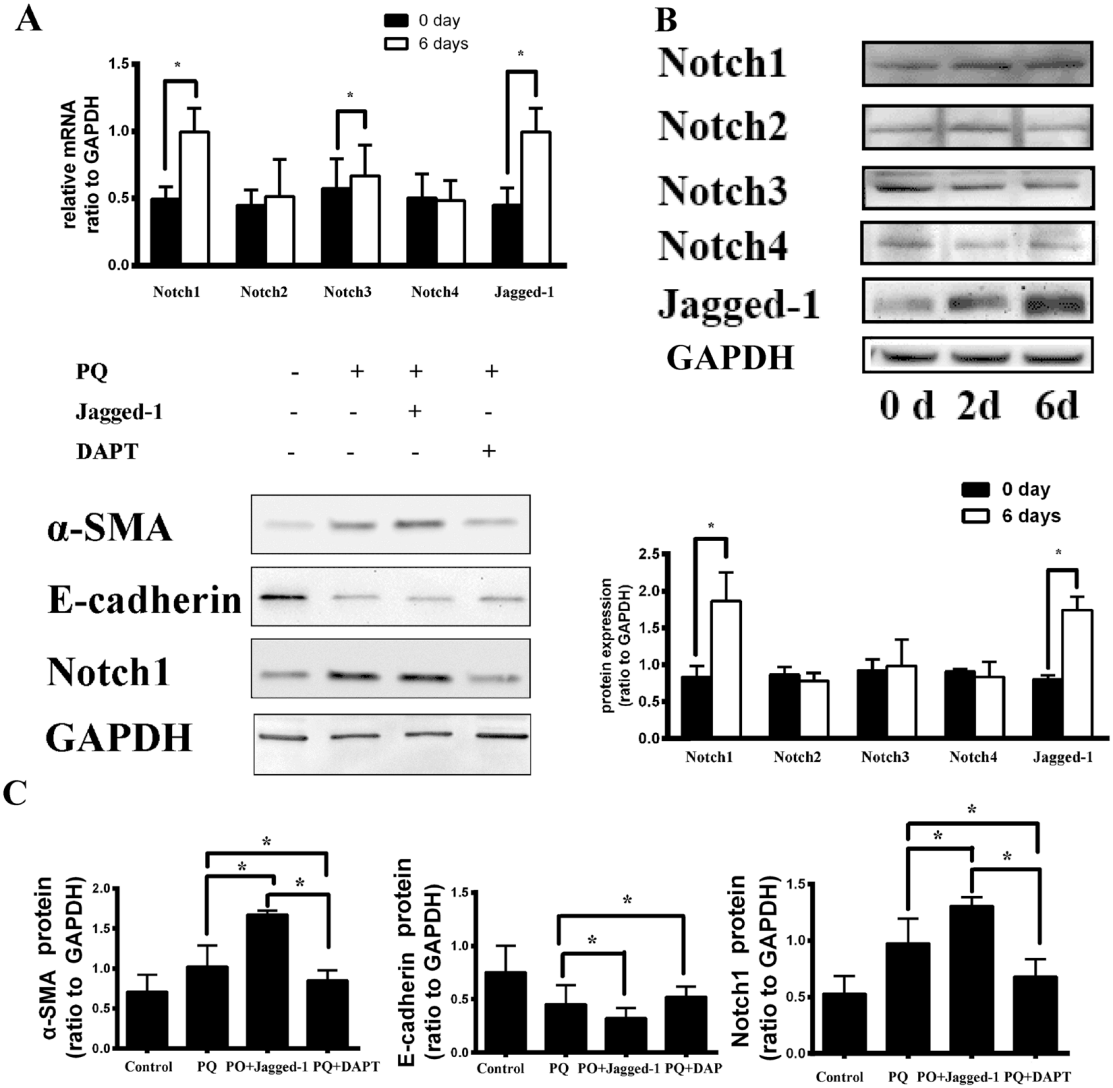
Notch1 signaling is required for PQ-induced EMT-like changes in A549 cells. (**A**) There was a significant increase in mRNA of Notch1, Notch3 and Jagged-1 after A549 cells were exposed to PQ for six days by qRT-PCR. (**B**) There was an apparent increase in expression of Notch1 and Jagged-1 protein by Western-blot analysis. (**C**) After treated with Jagged-1 ligand (ligand of Notch1, 500 ng/mL), a significant increase inα-SMA and Notch1, along with a decrease in E-cadherin protein by western analysis. However, after treating with DAPT (a γ-secretase inhibitor,10 μmol/L), α-SMA and Notch1 decreased than PQ and PQ + Jagged-1 group. *p < 0.05.

Next, we investigated whether Notch signaling plays an important role in PQ induced EMT process. We examined mRNA of Notch1–4 and Jagged-1 ligand by qRT-PCR analysis after A549 cells were exposed to PQ for six days. The results showed that there was a significant increase in mRNA of Notch1, Notch3 and Jagged-1 (p < 0.05), while the mRNA level of Notch2 and Notch4 did not change apparently (Fig. 2A). Western blot analysis, which was carried out to detect the changes in expression of all the proteins above, showed that there was an apparent increase in expression of Notch1 and Jagged-1. Notably, Notch3 protein expression did not increase, which was not consistent with the qRT-PCR result. A similar result with Notch2 and Notch4 protein expression were observed in comparison to mRNA expression using qRT-PCR(Fig. 2B).

Then, we added Jagged-1 ligand at a dose of 500 ng/mL or DAPT, a γ-secretase inhibitor, at a dose of 10 μmol/L, to A549 cells exposed to 300 μmol/L PQ for six days. Then we collected the cells and detected the expression of EMT markers, α-SMA and E-cadherin, and Notch1 pathway related proteins.

**Figure 3:**
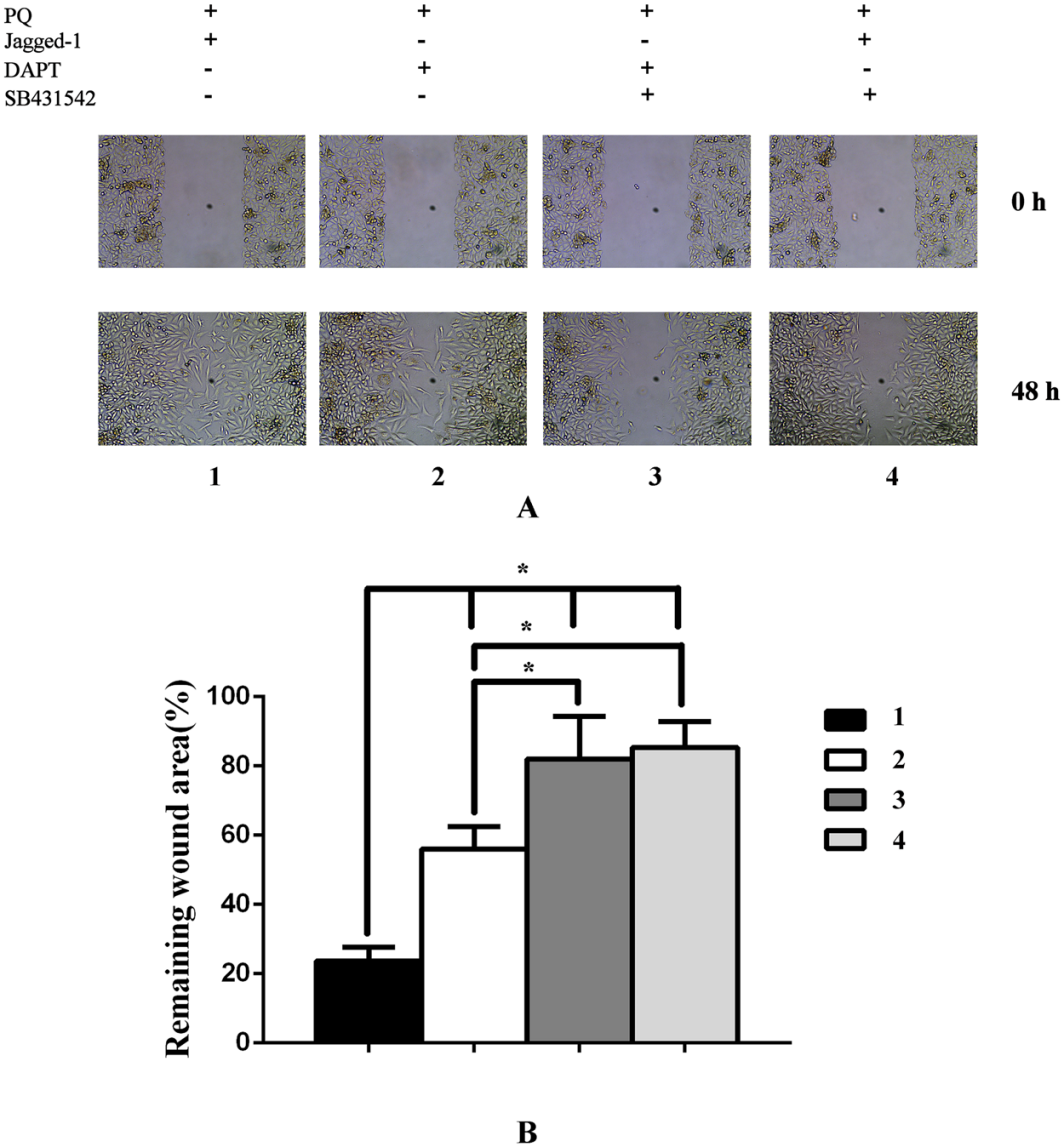
Monolayer wound-healing results of different treatments to A549 cells. A significantly increased migration capacity was observed after Jagged-1 (a Notch1 protein ligand, 500 ng/mL) stimulation, while a decreased capacity upon treated with DAPT (a γ-secretase inhibitor,10 μmol/L). And the migration property of A549 cells decreased apparently after treated with SB431542 (a specific inhibitor of TGF-β receptor type I/ALK5 kinase that phosphorylates Smad2/3, 10 μmol/L).

Upon stimulation with Jagged-1 ligand, we observed a significant increase in expression of α-SMA and Notch1, along with a decrease in E-cadherin protein by western blot analysis. However, after treated with DAPT, the relative amount of α-SMA and Notch1 decreased in comparison with PQ and PQ + Jagged-1 group (Fig. 2C). We also detected migration property of A549 cells in different conditions. A significantly increased migration capacity was observed after Jagged-1 stimulation, while a decreased capacity upon treated with DAPT (Fig. 3). The results revealed that Notch1 pathway is required in EMT induced by PQ poisoning, and it can be blocked by the γ-secretase inhibitor.

### PQ poisoning upregulates TGF-β/Smad3 pathway

TGF-β is implicated in the normal physiological repair as well as pathologic fibrotic processes of many organs, including the lungs^25, 26^. TGF-β is also implicated in EMT during the fibrosis in several tissues, including lungs^27^.

**Figure 4:**
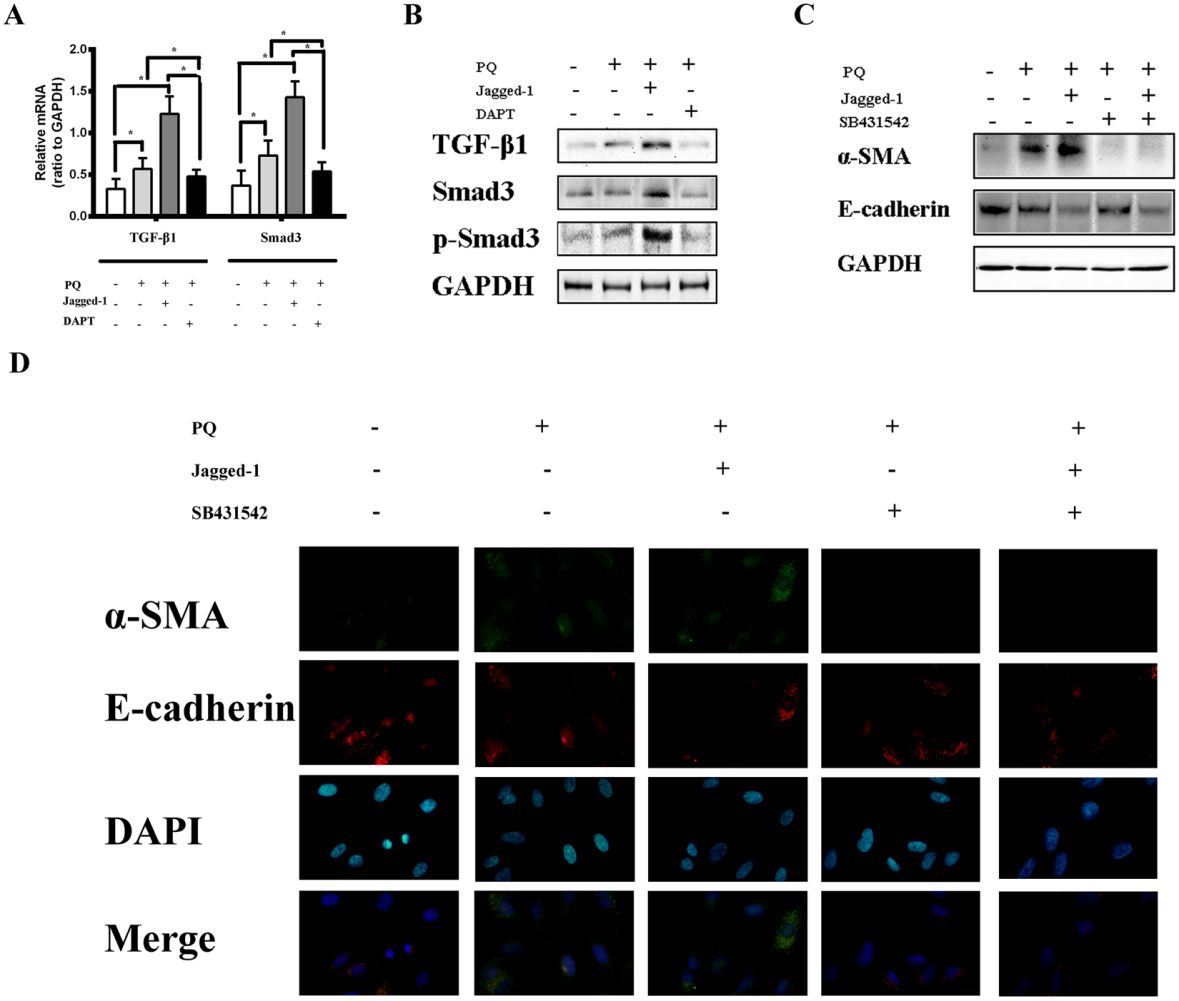
Notch1 pathway upregulates TGF-β1/Smad3 pathway. (**A**) We cultured A549 cells for 6 days, with PQ (300 μmol/L), PQ + Jagged-1(500 ng/mL), or PQ + DAPT (10 μmol/L). The mRNA level of TGF-β1 and Smad3 was increased after induction by Jagged-1, while DAPT could inhibit the mRNA of TGF-β1 and Smad3 byqRT-PCR. (**B**) A549 cells were cultured with PQ or PQ + Jagged-1, incubated in the presence or absence of SB431542 (a specific inhibitor of TGF-β receptor type I/ALK5 kinase that phosphorylates Smad2/3, 10 μmol/L) for 6 days. Jagged-1 significantly induced α-SMA, and this induction was completely inhibited by SB431542 in A549 cells by western blot analysis. (**C**) Cell membrane distribution of α-SMAand E-cadherin protein in A549 cells. Jagged-1 significantly induced expression of α-SMA protein, and this induction was inhibited by SB431542 in A549 cells. α-SMA was stained green, E-cadherin was stained red and DAPI stained nuclei blue. *p < 0.05.

So, in the following step, we investigated the expression of TGF-β1 in EMT caused by PQ. We analyzed TGF-β1/Smad3 after a six day culture of A549 cells with PQ, PQ + Jagged-1, or PQ + DAPT. The expression level of TGF-β1/Smad3 was increased after PQ poisoning, and was enhanced by Jagged-1, while DAPT could inhibit the mRNA of TGF-β1/Smad3 as demonstrated by qRT-PCR(Fig. 4A). Consistently, the result of western blot analysis indicated that Jagged-1 significantly increased the production of TGF-β1/Smad3, while DAPT decreased the expression of TGF-β1/Smad3 in A549 cells. In addition, the up-regulation of phosphorylated Smad3 protein was also induced by PQ alone and enhanced by Jagged-1 while blocked by DAPT in A549 cells (Fig. 4B).

To determine whether Smad phosphorylation is required for EMT induced by PQ poisoning, we utilized 10 μmol/L of SB431542, a specific inhibitor of TGF-β receptor type I/ALK5 kinase that phosphorylates Smad2/3. A549 cells were cultured with PQ or PQ + Jagged-1, incubated in the presence or absence of SB431542 (10 μmol/L) for six days, and then harvested for western blot analysis. Western blot analysis showed that Jagged-1 significantly induced α-SMA, and this induction was completely inhibited by SB431542 in A549 cells (Fig. 4C). Consistently, fluorescence immunostaining showed that SB431542 effectively prevented the increase in SMA expression induced by Jagged-1(Fig. 4D). Wound-healing assay also revealed apparently decreased migration behavior of A549 cells after treated with SB431542 (Fig. 3). Notably, expression of E-cadherin was not affected by SB431542. These results indicated that, in EMT caused by PQ, Notch1 acted through the induction of Smad3 phosphorylation.

## Discussion

PQ poisoning is a rising cause of death by insecticide poisoning, especially in developing countries. The mortality associated with PQ poisoning is increasing year by year. Most patients die from progressive pulmonary fibrosis following the acute phase of PQ poisoning. As there are no specific antidotes available as yet for PQ poisoning, in-depth investigation of the mechanism underlying progressive pulmonary fibrosis is utmost important. In this study, we exposed A549 cells to PQ and observed EMT-like phenotypic changes in A549 cells. Importantly, we demonstrate that Notch1 pathway is involved in EMT process after PQ poisoning, by up-regulating the expression of TGF-β/Smad3 signaling. This is the first investigation on the function of Notch pathway in EMT induced by PQ.

It is reported that PQ induces EMT during pulmonary fibrosis *in vitro* and *in vivo* ^13, 28^. In our study, we also observed EMT-like phenotypic changes in A549 cells. After exposure to PQ at a dose of 300 μmol/L for six days, we observed spindle-shaped changes in the A549 cells. The expression of α-SMA, a marker of mesenchymal cells, increased, while, E-cadherin, a marker of epithelial cells, decreased, as observed by qRT-PCR, western blotting and immunofluorescent staining. Subsequently, we tested the migration capacity of A549 cells, which is an important mobility change associated with EMT. An increased migration capacity of about 0.41-fold than negative control cells was observed upon PQ exposure. All the data above provided vital clues to suggest that A549 cells were transforming and acquiring characteristics and behavior of mesenchymal cells in response to PQ exposure.

Recent literature sheds light on the role of EMT, which occurs after PQ poisoning. However, the molecular mechanism of EMT induced by PQ poisoning is not yet established. To the best of our knowledge, our report is the first to demonstrate that Notch1 signaling is required for EMT induced by PQ poisoning. To explore the role of Notch1 in EMT process, we utilized Jagged-1 ligand to coactivate Notch1 signaling with PQ. Upon culture with Jagged-1, we observed changes in EMT-related markers such as α-SMA and E-cadherin which increased and decreased respectively. Meanwhile, when we used Notch1 signaling pathway inhibitor DAPT, the EMT process was blocked by the γ-secretase inhibitor. Migration capacity of A549 cells by wound healing assay was increased significantly after Jagged-1 stimulation, while decreased after DAPT treatment.

Subsequently, we proceeded to detect TGF-β, the known strongest inducer of fibrosis, in EMT after PQ exposure. Our results indicating TGF-β1 in response to PQ exposure suggest that the activation of TGF-β1 should be involved in EMT response. Moreover, the activation of TGF-β1 by Jagged-1 and suppression by DAPT indicated the essential induction role of Notch1 on TGF-β1. Furthermore, in PQ induced EMT, by using SB431542, a specific inhibitor of TGF-β receptor type I/ALK5 kinase that phosphorylates Smad2/3, we demonstrate that α-SMA induction by Jagged-1 can be completely inhibited. We provide evidence that the Notch signal acts upstream from the TGF-β1/Smad3 signal in EMT induced by PQ.

In our experiment, we demonstrated that Notch1/TGF-β1/Smad3 pathway is involved in the expression of α-SMA in EMT. However, the detailed functional point on α-SMA promoter zone is unknown, necessitating further advanced research on the α-SMA promoter.

## Conclusions

In conclusion, we demonstrated EMT-like phenotypic changes in A549 cells after PQ exposure. Notch1 signal was involved in the EMT process through TGF-β1/Smad3 signaling. This mechanism may contribute to the differentiation of myofibroblasts from alveolar epithelial cells during progressive pulmonary fibrosis caused by PQ poisoning.

## Methods

### Reagents

Paraquat dichloride hydrate, DAPT (N-(N-(3,5-difluorophenyl)-L-alanyl)-S-phenylglycine t-butylester) and SB431542 were purchased from Sigma-Aldrich (St. Louis, MO, USA). Recombinant human Jagged-1 Fc chimera protein was manufactured by R&D system (Minneapolis, MN, USA). Antibodies against NADPH, β-actin, TGF-β1 were purchased from Abcam (Cambridge, UK). Antibody against α-SMA and E-cadherin are provided by Santa Cruz Biotechnology (Dallas, TX, USA). Antibodies against Jagged-1, Notch1-4, Smad3 and phospho-Smad3 were purchased from Cell Signaling (Beverly, MA, USA).

### Cell culture and treatment

A549 human lung adenocarcinoma epithelial cells were cultured in DMEM medium supplemented with 10% fetal bovine serum, 100 U/mL penicillin, and 100 U/mL streptomycin at 37 °C in a humidified 5% CO_2_ atmosphere.

Before treatment, A549 cells were incubated in serum-free medium for 12 h. To evaluate the effects of Notch signal on EMT, 300 μmol/L PQ, 500 ng/mL recombinant Jagged-1 protein (a Notch1 protein ligand), and 10 μmmol/L DAPT (a γ-secretase inhibitor, 2 hour before stimulation by other reagents) were added to the cells. And 10 μmol/L SB431542, a specific inhibitor of TGF-β receptor type I/ALK5 kinase, were added to the cells at 2 hour before stimulation by other reagents. All the reagents need to be added again when changing medium.

### RNA Isolation and quantitative RT-PCR

Total cell RNA was isolated from A549 cells using the TRIzol reagent (Invitrogen, Carlsbad, CA, USA) according to the manufacturer's instructions. Fast SYBR Green Master Mix (Applied Biosystems, Foster City, CA, USA) and an ABI 7500 Fast real-time PCR instrument (Applied Biosystems, Warrington, UK) were used for quantitative real-time reverse transcription-PCR (qRT-PCR) with the gene-specific primer pairs listed in Table 1. The relative quantification results of gene expression were normalized to GAPDH transcript levels. For data analysis, the comparative threshold cycle (CT) value for GAPDH was used to normalize loading variations in the real-time PCRs.

**Table 1:** Primers sequence of reverse transcription-PCR analysis for genes.

Gene	Forward Primer	Reverse primer	Product length
*E-cadherin*	CGTAGCAGTGACGAATGTGGTAC	AACTGGAGAACCATTGTCTGTAGC	364
*α-SMA*	GGCTGTTTTCCCATCCATTGT	TCTTTTGCTCTGTGCTTCGT	103
*Notch1*	AGAGCTTTTCCTGTGTCTGTCC	CGGTACAGTCAGGTGTGTTGTT	414
*Notch2*	GACTGCCAATACTCGACCTC	TTCAGAAGTGAAGTCTCCAG	438
*Notch3*	CCTCTTTCACCTGTACCTGTCC	ACACAGTAGTGGGAGTGGTCCT	496
*Notch4*	CAACTCTGCGAGAACGGTGG	TGGAAGGAGCCCAAGGTGTT	443
*Jagged-1*	TCGCTGTATCTGTCCACCTG	AGTCACTGGCACGGTTGTAG	227
*TGF-β1*	TCCACCTGCAAGACTATCGAC	GAGGTATCGCCAGGAATTGTT	456
*Smad3*	ACCAGGGCTTTGAGGCTGTC	GCAAAGGCCCATTCAGGTG	144
*GAPDH*	GTCTCCTCTGACTTCAACAGCG	ACCACCCTGTTGCTGTAGCCAA	131

### Western blot analysis

Following exposure to PQ, the cell lysates were prepared at the indicated time points using RIPA buffer. Protein concentration was determined by the Bradford assay. Equal amounts of proteins were transferred to a nitrocellulose membrane. After blocking, the membrane was incubated overnight at 4 °C with appropriate primary antibodies. Anti-E-cadherin, anti-α-SMA, anti-Notch1-4, anti-TGF-β1, anti-Smad3 and anti-phospho-Smad3 polyclonal antibodies were used at 1/1000 dilution. Then the membrane was washed with TBS-Tween and incubated with peroxidase-conjugated secondary antibodies at 1/4000 dilution. Immunoreactive proteins were visualized using enhanced chemiluminescence western blotting detection reagents and detected by HMIAS-2000 Imaging System. Band densities were determined by BioRad Quantity One software and quantified as the ratio of GAPDH protein expression.

### Monolayer wound-healing assay

After exposed to PQ for 6 days, A549 cells were collected for monolayer wound-healing assay. Briefly, A549 cells were grown in a 6-well plate, and linear wounds were made in the confluent monolayer using a 20 μL pipette tip. In each experiment, one well was used as a negative control with no treatment. The wounds were imaged 0 and 48 hours after wound creation. Corresponding wound areas were determined using Image-J and the remaining wound areas were calculated as a percentage of area at time 0^29, 30^.

### Fluorescence immunostaining

A549 cells grown on cover slips were fixed with 4% paraformaldehyde for 10 min room temperature and stained with primary antibody: anti-E-cadherin or anti-α-SMA overnight at 4 °C, followed by the secondary antibody. Nuclei were stained with 4, 6-diamidino-2-phenylindole (DAPI). Fluorescence images were observed under a fluorescence microscope.

### Statistical Analysis

Data are presented as the mean ± standard deviation. The statistically significant difference was analyzed by independent sample *t* test for two groups, and ANOVA for three and more groups. SPSS16.0 software was used for statistical analysis. P < 0.05 was considered as statistically significant.

## References

[1] Sabzghabaee AM, Eizadi-Mood N, Montazeri K, Yaraghi A, Golabi M (2010). Fatality in paraquat poisoning. Singapore medical journal.

[2] Suntres ZE (2002). Role of antioxidants in paraquat toxicity. Toxicology..

[3] Phan SH (2008). Biology of fibroblasts and myofibroblasts. Proc. Am. Thorac. Soc.

[4] Nakashima T (2013). Lung bone marrow-derived hematopoietic progenitor cells enhance pulmonary fibrosis. Am. J. Respir. Crit. Care. Med..

[5] Kisseleva T, Brenner DA (2008). Mechanisms of fibrogenesis. Exp. Biol. Med. (Maywood).

[6] Kalluri R, Neilson EG (2003). Epithelial-mesenchymal transition and its implications for fibrosis. J. Clin. Invest..

[7] Acloque H, Adams MS, Fishwick K, Bronner-Fraser M, Nieto MA (2009). Epithelial-mesenchymal transitions: the importance of changing cell state in development and disease. J. Clin. Invest..

[8] Kalluri R, Weinberg RA (2009). The basics of epithelial-mesenchymal transition. J. Clin. Invest..

[9] Thiery JP, Acloque H, Huang RY, Nieto MA (2009). Epithelial-mesenchymal transitions in development and disease. Cell..

[10] Chapman HA (2011). Epithelial-mesenchymal interactions in pulmonary fibrosis. Annu. Rev. Physiol..

[11] Kage H, Borok Z (2012). EMT and interstitial lung disease: a mysterious relationship. Curr. Opin. Pulm. Med..

[12] Sheppard D (2015). Epithelial-mesenchymal interactions in fibrosis and repair. Transforming growth factor-beta activation by epithelial cells and fibroblasts. Ann. Am. Thorac. Soc..

[13] Yamada A, Aki T, Unuma K, Funakoshi T, Uemura K (2015). Paraquat induces epithelial-mesenchymal transition-like cellular response resulting in fibrogenesis and the prevention of apoptosis in human pulmonary epithelial cells. PLoS. One..

[14] Bray SJ (2006). Notch signalling: a simple pathway becomes complex. Nat. Rev. Mol. Cell. Biol..

[15] Koch U, Lehal R, Radtke F (2013). Stem cells living with a Notch. Development..

[16] Jarriault S (1995). Signalling downstream of activated mammalian Notch. Nature..

[17] Du R (2012). Hypoxia-induced down-regulation of microRNA-34a promotes EMT by targeting the Notch signaling pathway in tubular epithelial cells. PLoS. One..

[18] Kobayashi T (2008). Expression and function of the Delta-1/Notch-2/Hes-1 pathway during experimental acute kidney injury. Kidney. Int..

[19] Nijjar SS, Wallace L, Crosby HA, Hubscher SG, Strain AJ (2002). Altered Notch ligand expression in human liver disease: further evidence for a role of the Notch signaling pathway in hepatic neovascularization and biliary ductular defects. Am. J. Pathol..

[20] Walsh DW (2008). Co-regulation of Gremlin and Notch signalling in diabetic nephropathy. Biochim. Biophys. Acta..

[21] Waters AM (2008). Ectopic notch activation in developing podocytes causes glomerulosclerosis. J. Am. Soc. Nephrol..

[22] Liu T (2004). FIZZ1 stimulation of myofibroblast differentiation. Am. J. Pathol..

[23] Zavadil J, Cermak L, Soto-Nieves N, Bottinger EP (2004). Integration of TGF-beta/Smad and Jagged1/Notch signalling in epithelial-to-mesenchymal transition. EMBO. J..

[24] Aoyagi-Ikeda K (2011). Notch induces myofibroblast differentiation of alveolar epithelial cells via transforming growth factor-{beta}-Smad3 pathway. Am. J. Respir. Cell. Mol. Biol..

[25] Bartram U, Speer CP (2004). The role of transforming growth factor beta in lung development and disease. Chest..

[26] Willis BC, Borok Z (2007). TGF-beta-induced EMT: mechanisms and implications for fibrotic lung disease. Am. J. Physiol. Lung. Cell. Mol. Physiol..

[27] Xaubet A (2003). Transforming growth factor-beta1 gene polymorphisms are associated with disease progression in idiopathic pulmonary fibrosis. Am. J. Respir. Crit. Care. Med..

[28] Xie H (2013). Expression and significance of HIF-1alpha in pulmonary fibrosis induced by paraquat. Exp. Biol. Med. (Maywood).

[29] Allahverdian S (2008). Secretion of IL-13 by airway epithelial cells enhances epithelial repair via HB-EGF. Am. J. Respir. Cell. Mol. Biol..

[30] Itoigawa Y (2015). TWEAK enhances TGF-beta-induced epithelial-mesenchymal transition in human bronchial epithelial cells. Respir. Res..

